# Influence of the timing of biological treatment initiation on Juvenile Idiopathic Arthritis long-term outcomes

**DOI:** 10.1186/s13075-023-03166-9

**Published:** 2023-09-21

**Authors:** Filipa Oliveira Ramos, Ana Maria Rodrigues, Ana Teresa Melo, Francisca Aguiar, Luísa Brites, Soraia Azevedo, Ana Catarina Duarte, José António Melo Gomes, Carolina Furtado, Ana Filipa Mourão, Graça Sequeira, Inês Cunha, Ricardo Figueira, Maria José Santos, João Eurico Fonseca

**Affiliations:** 1 Pediatric Rheumatology Unit, Hospital Santa Maria, Centro Hospitalar Universitário Lisboa Norte, Lisbon, Portugal; 2 Unidade de Investigação Em ReumatologiaInstituto de Medicina Molecular, Lisbon, Portugal; 3 Faculdade de Medicina, Universidade de Lisboa, Lisbon Academic Medical Center, Lisbon, Portugal; 4 Centre for Chronic Diseases (CEDOC), Nova Medical School, Lisbon, Portugal; 5 Comprehensive Health Research Centre, Nova Medical School, Lisbon, Portugal; 6 Young Adult and Pediatric Rheumatology Unit, Centro Hospitalar Universitário São João, University of Medicine of Porto University, Porto, Portugal; 7 Rheumatology Department, Centro Hospitalar E Universitário de Coimbra, Coimbra, Portugal; 8 Rheumatology Department, Unidade Local de Saúde Do Alto Minho, Ponte de Lima, Portugal; 9 Rheumatology Department, Hospital Garcia de Orta, Almada, Portugal; 10 Instituto Português de Reumatologia, Lisbon, Portugal; 11 Rheumatology Department, Hospital Do Divino Espírito Santo, Ponta Delgada, Portugal; 12 Rheumatology Department, Centro Hospitalar de Lisboa Ocidental, Lisbon, Portugal; 13 Rheumatology Department, Centro Hospitalar Universitário Do Algarve, Faro Unit, Faro, Portugal; 14 Rheumatoloy Department, Centro Hospitalar Do Baixo Vouga, Aveiro, Portugal; 15 Rheumatoloy Department, Hospital Central Do Funchal, Funchal, Portugal

**Keywords:** Juvenile idiopathic arthritis, Long-term outcomes, bDMARDs, Physical disability, Quality of life

## Abstract

**Background:**

Juvenile idiopathic arthritis (JIA) treatment is aimed at inducing remission to prevent joint destruction and disability. However, it is unclear what is the long-term impact on health-related outcomes of the timing of biological disease-modifying antirheumatic drug (bDMARD) initiation in JIA. Our aim was to evaluate the long-term impact of the time between JIA onset and the initiation of a bDMARD in achieving clinical remission, on physical disability and health-related quality of life (HRQoL).

**Methods:**

Adult JIA patients registered in the Rheumatic Diseases Portuguese Register (Reuma.pt) and ever treated with bDMARD were included. Data regarding socio-demographic, JIA-related characteristics, disease activity, physical disability (HAQ-DI), HRQoL (SF-36), and treatments were collected at the last visit. Patients were divided into 3 groups (≤ 2 years, 2–5 years, or > 5 years), according to the time from disease onset to bDMARD initiation. Regression models were obtained considering remission on/off medication, HAQ-DI, SF-36, and joint surgeries as outcomes and time from disease onset to bDMARD start as an independent variable.

**Results:**

Three hundred sixty-one adult JIA patients were evaluated, with a median disease duration of 20.3 years (IQR 12.1; 30.2). 40.4% had active disease, 35.1% were in remission on medication, and 24.4% were in drug-free remission; 71% reported some degree of physical disability. Starting a bDMARD > 5 years after disease onset decreased the chance of achieving remission off medication (OR 0.24; 95% CI 0.06, 0.92; *p* = 0.038). Patients who started a bDMARD after 5 years of disease onset had a higher HAQ and worse scores in the physical component, vitality, and social function domains of SF-36, and more joint surgeries when compared to an earlier start.

**Conclusion:**

Later initiation of bDMARDs in JIA is associated with a greater physical disability, worse HRQoL, and lower chance of drug-free remission in adulthood.

## Introduction

The term juvenile idiopathic arthritis (JIA) is used to designate a very heterogeneous group of chronic inflammatory diseases with childhood onset that actually correspond to distinct diseases with different prognoses [1]. In the current era of individualized treat-to-target treatment strategies [2], knowledge about the long-term outcomes of patients with JIA is essential for patient counseling and planning the transition to adult care. Since 1999, the introduction of biologics in JIA treatment has dramatically changed the long-term functional outcome of JIA, and over the last decade, the outcomes of JIA patients have been studied in cohorts with progressively longer follow-up [3–7]. Some studies have shown that the sooner treatment is begun for JIA and the more aggressive it is, the better the outcomes obtained [8–11]. However, it is less clear what is the long-term impact of the timing of biological disease-modifying antirheumatic drug (bDMARD) initiation in JIA. This timing is affected by multiple factors like the number of active joints, presence and severity of uveitis, and JIA category [12], and for this reason, the optimum time for initiating a bDMARD is difficult to establish as it varies greatly among patients.

This study aims to evaluate the long-term impact in adulthood of the time between the onset of JIA and the initiation of a bDMARD on achieving clinical remission, on physical disability, and on health-related quality of life (HRQoL).

## Methods

### Study design and patient selection

This is a cross-sectional analysis nested in a cohort study with the following inclusion criteria: patients with JIA according to the 2001 revised International League of Associations for Rheumatology (ILAR) criteria [1], registered in the Rheumatic Diseases Portuguese Register (Reuma.pt) [13], who at the time of data extraction (September 2021) were older than 18 years old, had ever been treated with bDMARD and have available data in adulthood.

Reuma.pt was developed by the Portuguese Society of Rheumatology, became active in June 2008, and includes patients with JIA, rheumatoid arthritis (RA), spondyloarthritis (SpA), and several other rheumatic diseases. Specifically, 2081 JIA patients with 18856 medical visits have been registered so far in Reuma.pt [14]. Data before 2008 was registered retrospectively and prospectively thereafter. Patients with disease onset before 2001 were classified retrospectively according to the ILAR classification.

Registry of patient data in Reuma.pt occurred after signed informed consent was obtained. This study was approved by the scientific committee of Reuma.pt and by the ethics committee of the Lisbon Academic Medical Centre. Reuma.pt was approved by the National Data Protection Authority and by local ethics committees of the participating centers. The study was conducted according to the Declaration of Helsinki.

### Clinical data collection

The following information was obtained from the last visit available at the moment of data extraction (September 2021): sex, ethnicity, age at last visit, years of education, employment status (employed, unemployed, retired and retired due to JIA induced disability), ILAR category, age at disease onset (disease onset was defined as the date when arthritis was first documented by a physician), disease duration (years), presence of rheumatoid factor (RF), anti–citrullinated protein antibodies (ACPA), antinuclear antibodies (ANA); considered positive if titers ≥ 1/160) and human leukocyte antigen B27 (HLA B27), number of active joints, patient and physician’s global assessment of disease activity (0–10), back pain (0–10), morning stiffness intensity (0–10), erythrocyte sedimentation rate (mm/first hour) and C-reactive protein level (mg/dl), extra-articular manifestations, joint surgeries (surgical synovectomy, arthroplasty, and arthrodesis), Juvenile Arthritis Damage Index (JADI) score, current and previous therapy with corticosteroids, conventional disease modifying antirheumatic drugs (cDMARD), and bDMARDs.

Assessment of the disease activity was based on the American College of Rheumatology (ACR) provisional criteria [15] for clinical inactive disease (CID) and clinical remission (CR). Remission off drugs was defined as CID for at least 12 months without any treatment, in accordance with the criteria by Wallace et al. [16].

In the absence of a validated score for evaluation of damage in adults with JIA, we opted to use JADI, as a more comprehensive way of assessing articular damage (JADI-A) and extra-articular damage (JADI-E), [17].

The physical disability was measured by the Health Assessment Questionnaire—Disability Index (HAQ-DI), [18] obtained at the last visit registered. For the purpose of this analysis, mild disability was considered for HAQ scores > 0 and ≤ 0.5, moderate disability > 0.5 and ≤ 1.5, and severe disability > 1.5 [19].

HRQoL was assessed using the Medical Outcomes Study 36-item Short Form (SF-36) [20].

### Statistical analysis

Descriptive statistics were presented through medians and interquartile range (IQR) due to the absence of Gaussian distribution evaluated by the Shapiro–Wilk test. Qualitative data were presented through absolute and relative frequencies. The former statistics were compared according to the time when the first biologic was started (< 2 years; 2–5 years; > 5 years after disease onset) using, respectively, the Kruskal–Wallis test with pairwise adjusted comparisons when justified, and the Fisher exact test. Afterwards, the variable “time when the first biologic was started” was recoded into two groups, (≤ 5 years; > 5 years) as most of the statistically significant differences were between the ones who started the first biologic for more than 5 years and one or both of the other two groups, as binary variables are more easily interpreted than variables with three or more categories, even when ordinal. These groups were compared through the Mann–Whitney* U* test or the Fisher exact test, according to previous considerations. This step was performed in order to select meaningful variables for further analysis.

Binary logistic and linear regression was then applied, considering joint surgeries, disease activity, physical disability, and quality of life as dependent variables, and considering the “time when the first biologic was started” as an independent variable. We performed two adjusted models to determine the association between each of the health-related outcomes (joint surgeries, clinical remission on and off medication, physical disability, and quality of life) with “time when the first biologic was started.” The first model was adjusted for gender and JIA categories and the second model adjusted for gender, JIA categories, disease duration and disease activity (for the outcomes remission on and off medication adjustment for disease activity was not applied). Due to the high variability of some of the covariates it is of extreme importance to state that models presented white noise residuals with mean zero, that is, residuals presented normal distribution — evaluated through the Shapiro–Wilk test — with mean zero and constant variance, leading to homogeneity of residuals according to predictions. Independency and absence of autocorrelation were evaluated and confirmed by the Durbin-Watson statistic.

Missing data were interpreted as random missing data without any data imputation. Analysis was performed in SPSS, version 27, and evaluated at a 5% significant level.

## Results

### Patient characteristics

**Fig. 1:**
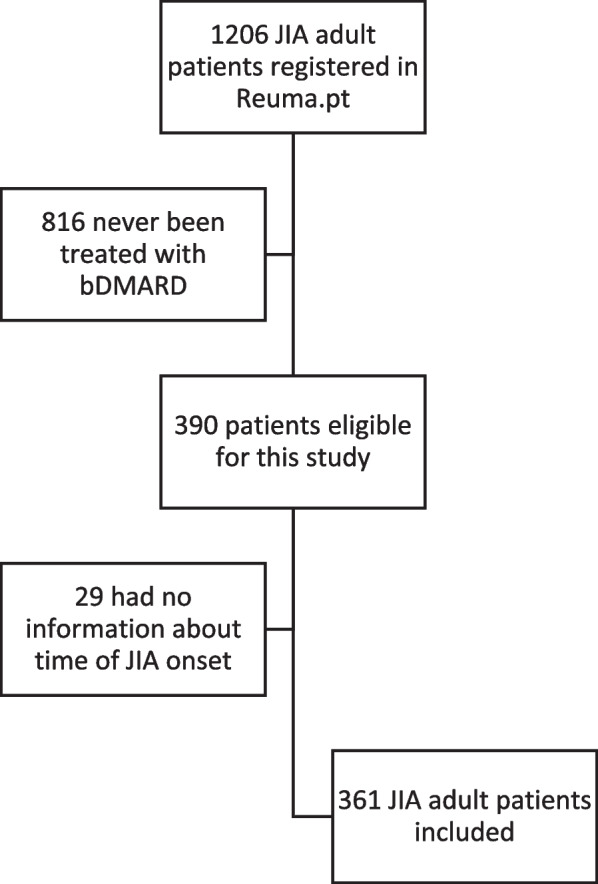
Disposition of adult JIA patients registered in Reuma.pt eligible for this study

A total of 361 adult JIA patients who had ever been treated with a bDMARD were included in the study (Fig. 1). From these 361 patients, 279 patients were diagnosed previously to 2008 when Reuma.pt became active and had data registered retrospectively until that year and prospectively afterward. For 82 patients all the data was registered prospectively. The 226 patients who had their disease onset before 2001 were classified retrospectively according to the ILAR classification. The main demographic and clinical features of the patients included in this study are shown in Table 1.

**Table 1:** Socio-demographic and disease-related characteristics of JIA patients according to the time when biologic was started

	Total	bDMARD started < 2 years after disease onset	bDMARD started 2–5 years after disease onset	bDMARD started > 5 years after disease onset	*p*-value
Patients, *n* (%)	361 (100)	52 (14.4)	65 (18)	244 (67.5)	
Female, *n* (%)	194 (53.7)	19 (36.5)	21 (32.3)	154 (63.1)^c^	0.015*
**JIA ILAR category**, *n* (%)					0.001**
Persistent oligoarthritis	33 (9.1)	2 (3.8)	12 (18.5)	19 (7.8)	
Extended oligoarthritis	58 (16.1)	7 (13.5)	8 (12.3)	43 (17.6)	
RF-positive polyarthritis	39 (10.8)	3 (5.8)	4 (6.2)	32 (13.1)	
RF-negative polyarthritis	70 (19.4)	10 (19.2)	11 (16.9)	49 (20.1)	
Systemic	34 (9.4)	5 (9.6)	8 (12.3)	21 (8.6)	
Enthesitis related arthritis	88 (24.4)	22 (42.3)	16 (24.6)	50 (20.5)	
Psoriatic arthritis	17 (4.7)	3 (5.8)	4 (6.2)	10 (4.1)	
Undifferentiated arthritis	22 (6.4)	0 (0)	3 (4.6)	19 (7.8)	
Age at disease onset, years, median (IQR)	11.5 (6.3–14.6)	13.3 (11.7–15.7)^a^	12.2 (9.8–14.7)^b^	9.8 (5.3–14)^a,b^	< 0.001*
Age at diagnosis, years, median (IQR)	13.3 (8.2–16.8)	13.1 (10.4–16.1)	13.0 (10.6–15.7)	13.2 (6.8–18.2)	0.392
Age when first biologic was started, years, median (IQR)	21.7 (15.8–31.9)	15.3 (13.4–16.4)^a^	15.8 (13–17.9)^b^	26.3 (19.7–36)^a,b^	< 0.001*
Disease duration, years, median (IQR)	20.3 (12.1–30.2)	7.5 (3.8–11.6)^a^	10.8 (6.4–15.9)^b^	25.1 (19.3–34.1)^a,b^	< 0.001*
Age at last visit, years, median (IQR)	29.1 (21.8–40.2)	20.2 (18.9–23.2)^a^	21.9 (19.7–25.5)^b^	35.5 (27–44.8)^a,b^	< 0.001*
ANA + , *n* assessed: 225, *n* (%)	71 (31.5)	9 (21.4)	11 (25.6)	51 (36.4)^c^	0,003*
RF + , *n* assessed: 267, *n* (%)	73 (27.3)	8 (20)	5 (11.1)	60 (33)^c^	0.032*
ACPA + , *n* assessed: 175, *n* (%)	47 (26.8)	7 (22.5)	3 (10.3)	37 (32.2)	0.271
HLAB27 + , *n* assessed: 183, *n* (%)	78 (42.6)	17 (48.6)	14 (45.2)	47 (40.2)	0.164
Presence of uveitis, *n* assessed: 285, *n* (%)	40 (14)	2 (5)	9 (20)	29 (14.5)	0.105
Years of education, *n* assessed: 203; median (IQR)	12 (9–15)	11 (9–12)	11 (9–14)	12 (9–15)^c^	0.24
**Professional situation**, *n* assessed: 210					0.193
Employed, *n* (%)	159 (75.7)	18 (60)^c^	21 (63.6) ^c^	120 (81.6)	
Unemployed, *n* (%)	18 (8.6)	0 (0)	1 (3)	17 (11.6)^c^	
Retired, *n* (%)	7 (3.3)	0 (0)	0 (0)	7 (4.8)^c^	
Retired due to JIA disability, *n* (%)	17 (8.1)	0 (0)	0 (0)	17 (11.6)^c^	
**Past treatment**					
Corticosteroids, *n* (%)	181 (50.1)	21 (40.4)	15 (23.1)	145 (59.4)^c^	0.002
cDMARDs, *n* (%)	280 (77.6)	34 (65.4)	38 (58.5)	208 (85.2)	0.709
**Current treatment**					
Corticosteroids, *n* assessed: 271, *n* (%)	88 (32.5)	4 (14.3)	3 (7.3)	81 (40.1)^c^	< 0,001
cDMARDs, *n* assessed: 272, *n* (%)	159 (58.5)	13 (44.8)	15 (36.6)	129 (63.9)	0.148
bDMARDS, *n* (%)	295 (81.7)	33 (63.5)	36 (55.4)	196 (80.3)	0.817
Cumulative corticosteroid exposure, years, median (IQR)	5.9 (2.4–14.9)	1.8 (0.8–2.3)^a^	3.9 (1.9–8.2)	7.8 (2.9–17.1]^a^	< 0.001*

Sample size is not constant due to missing data:

Patients whose bDMARD was started < 2 years after disease onset: ANA + *n* = 42; RF + *n* = 40; ACPA + *n* = 31; HLAB27 + *n* = 35; presence of uveitis *n* = 40; professional situation *n* = 30; current corticosteroids *n* = 28; current cDMARDs n = 29

Patients whose bDMARD was started 2–5 years after disease onset: ANA + *n* = 43; RF + *n* = 45; ACPA + *n* = 29; HLAB27 + *n* = 31; presence of uveitis *n* = 45; professional situation *n* = 33; current corticosteroids *n* = 41; current cDMARDs *n* = 41

Patients whose bDMARD was started > 5 years after disease onset: ANA + *n* = 140; RF + *n* = 182; ACPA + *n* = 115; HLAB27 + *n* = 117; presence of uveitis *n* = 200; professional situation *n* = 147; current corticosteroids *n* = 202; current cDMARDs *n* = 202

*Legend*: *JIA* juvenile idiopathic arthritis, *IQR* interquartile range, *ANA* antinuclear antibodies, *RF* rheumatoid factor, *ACPA* anti-citrullinated protein antibodies, *DMARD* disease-modifying antirheumatic drugs, *cDMARDs* conventional DMARD, *bDMARDs* biological DMARDs

^a^Statistically significant difference between “ < 2Y” and “ > 5Y” at a 5% significance level — Mann–Whitney* U* test adjusted for multiple comparisons

^b^Statistically significant difference between “2–5Y” and “ > 5Y” ate a 5% significance level — Mann–Whitney* U* test adjusted for multiple comparisons

^c^Higher frequency than expected (statistically significant at a 5% significance level — Fisher exact test and adjusted residual higher than 1.96)

^*^Statistically significant difference between “ < 5Y” and “ ≥ 5Y” at a 5% significance level — Mann–Whitney* U* test

^**^Fisher exact test applied using “Extended oligoarthritis,” “RF-positive polyarthritis,” and “RF-negative polyarthritis” categories of JIA grouped as one in order to perform the test with some robustness

The median age at the last registered visit was 29.1 years (IQR 21.8–40.2; range: 18–74) and the median disease duration was 20.3 years (IQR: 12.1–30.2; range: 1–71.5). Most of the patients (79%) had a disease duration of more than 10 years and 24% exceeded 30 years.

The distribution of the JIA categories over the 3 different timings of bDMARD initiation groups (< 2 years; 2–5 years; > 5 years) is shown in Table 1.

The first bDMARD treatment was started in 14.4% within two years of JIA onset, 18% after two to five years of disease, and 67.5% more than 5 years after disease onset. The median age at bDMARD start was 15.5 years (IQR 13.4–16.5) in patients that started this treatment in the first 2 years after disease onset and 15.7 years (IQR 13.1–17.8) in the group that started bDMARD 2–5 years after disease onset. The patients who started bDMARD after 5 years of disease onset had a median age at bDMARD start of 26.28 years (IQR 19.7–35.6), were older at the last visit than the patients from the other groups, and had longer disease duration, and 72% had the disease onset before the year 2000. In the subgroup of this cohort that had the disease onset after the year 2000 (*n* = 169) the distribution according to the 3 groups was 29.4% in both groups that started bDMARD before 5 years after disease onset and 41.6% in the group that started later, after 5 years of disease onset.

Forty percent of the studied patients still had active disease and 80.6% were on a cDMARD or bDMARD. The most frequently used cDMARD was methotrexate in 70.9% of the cases and the most frequently used bDMARD was etanercept in 54.8% followed by adalimumab in 29.6% of the cases. Only 14.9% of the total cohort was in remission off medication. 61.5% of the patients had no or mild disability and just 12.9% had severe disability. Cumulative corticosteroid exposure was significantly higher in the > 5 years group when compared to the patients who started bDMARD earlier in the disease course (7.8 [IQR 2.9–17.1] vs 3.9 [IQR 1.9–8.2] and 1.8 [IQR 0.8–2.9]; *p* < 0.001).

### Patient health-related outcomes according to the timing of bDMARD initiation

Patients who started bDMARD after 5 years of disease onset were more likely to have active disease when compared to the patients who started bDMARD earlier (85.7% vs 8.8% and 5.5.% respectively, *p* < 0.001; Table 2). This group of patients with a later start of bDMARD was also 76% less likely to be on remission off medication, irrespectively of JIA category, gender, and disease duration (OR 0.24; 95% CI 0.06, 0.92; *p* = 0.038; Table 3).

**Table 2:** Outcomes at the last follow-up according to the time when bDMARD was started

	Total	bDMARD started < 2 years after disease onset	bDMARD started 2–5 years after disease onset	bDMARD started > 5 years after disease onset	*p*-value
**Disease activity** (n assessed: 225)					
Active (*N*/%)	91 (40.4)	5 (5.5)	8 (8.8)	78 (85.7)^c^	< 0.001*
Remission on medication (*N*/%)	79 (35.1)^c^	10 (12.6)	12 (15.1)	57 (72.1)	0.156
Remission off medication (*N*/%)	54 (24)^c^	8 (14.8)^c^	14 (25.9)	32 (59.2)	< 0.001*
**JADI-A** (*n* assessed: 158; median (IQR))	1 (0–14.5)	0 (0–1)^a^	0 (0–1)^b^	3 (0–28)^a,b^	0.002*
**JADI-E** (*n* assessed: 158; median (IQR))	0 (0–1)	0 (0–0)^a^	0 (0–0)	0 (0–2)^a^	0.013*
**HAQ** (*n* assessed: 283; median (IQR))	0.25 (0–1)	0 (0–0.25)^c^	0 (0–0.09)^b^	0.5 (0–1.25)^a,b^	< 0.001*
**SF36 physical component** (*n* assessed: 218; median (IQR))	41.9 (29.9–53.2)	56.2 (44.2–58.6)^c^	49.5(44.0–57.8)^b^	40.2 (32.9–64.4)^a,b^	0.001
SF36 PF (median (IQR))	67.5 (40–95)	97.5 (82.5–100)	92.5 (70–100)^b^	55 (30–90)^a,b^	< 0.001*
SF36 RP (median (IQR))	93.8 (25–100)	100 (64.1–100)	100 (87.5–100)^b^	75 (25–100)^b^	0.013*
SF36 BP (median (IQR))	62 (41–84)	73 (38.8–88)	82 (61.3–96)^b^	52 (41–84)^b^	0.019*
SF36 GH (median (IQR))	47 (32.8–67)	73,5 (50.8–87)^c^	45 (37–67)	47 (30–67)^a^	0.046
**SF36 mental component** (*n* assessed: 217; median (IQR))	48.1 (37.3–65.8)	54.0 (32.9–64.4)	58.4 (43.4–72.1)	47.4 (34.7–66.6)	0.165
SF36 VT (median (IQR))	50 (40–75)	65.6 (50–82.2)	63.8 (55.3–79.7)^b^	50 (40–69.4)^b^	0.008*
SF36 SF (median (IQR))	87.5 (51–100)	87.5 (71.9–100)	100 (78.1–100)^b^	75 (50–100)^b^	0.017*
SF36 RE (median (IQR))	100 (64.6–100)	100 (91.7–100)	100 (100–100)	100 (33.3–100)	0.154
SF36 MH (median (IQR))	76 (60–88)	86.5 (77.5–91.3)	75 (61.4–90)	76 (60–88)	0.198
**Joint surgery ever** (*n* assessed: 361; *N*/%)	61 (16.9)	0 (0.0)	1 (2.2)	57 (24.1)	< 0.001*

*Legend*: *IQR* interquartile range, *bDMARD* biological disease-modifying antirheumatic drugs, *JADI-A* Juvenile Arthritis Damage Index – articular, *JADI-E* Juvenile Arthritis Damage Index – extra-articular, *HAQ* Health Assessment Questionnaire, *SF36* Medical Outcomes Study 36-item Short Form, *SF36 PC* physical component of the Short Form 36, *SF36 MC* mental component of the Short Form 36, *PF* physical function, *RP* role limitations due to physical problems, *BP* intensity and discomfort caused by pain, *GH* general health, *VT* vitality, *SF* social function, *RE* role limitations due to emotional problems, *MH* mental health

^a^Statistically significant difference between “ < 2Y” and “ > 5Y” at a 5% significance level — Mann–Whitney* U* test adjusted for multiple comparisons

^b^Statistically significant difference between “2–5Y” and “ > 5Y” ate a 5% significance level — Mann–Whitney* U* test adjusted for multiple comparisons

^c^Higher frequency than expected (Statistically significant at a 5% significance level — Fisher exact test and adjusted residual higher than 1.96)

^*^Statistically significant difference between “ < 5Y” and “ ≥ 5Y” at a 5% significance level — Mann–Whitney* U* test

**Table 3:** Multivariate regression to identify the association of the timing of bDMARD start and remission on and off medication, physical disability, HRQoL, and joint surgeries as outcomes

**Outcomes**	** > 5 years from disease-onset to bDMARD start, adjusted for JIA category**^c^** and gender**		** > 5 years from disease-onset to bDMARD start, adjusted for JIA category**^c^**, gender, disease duration, and disease activity**	
	Beta/OR (95% CI)	*p*-value	Beta/OR (95% CI)	*p*-value
**Remission on medication**^a^	0.61 (0.25; 1.49)	0.278	2.47 (0.55; 11.12)^d^	0.240
**Remission off medication**^a^	**0.28 (0.12; 1.66)**	**0.003**	**0.24 (0.06; 0.92)**^d^	**0.038**
**HAQ total score**^b^	**0.48 (0.27; 0.68)**	** < 0.001**	0.05 (− 0.18; 0,28)	0.687
**SF 36 physical component**^b^	** − 10.63 (− 16.00; − 5.27)**	** < 0.001**	− 0.01 (− 8.76; 6.85)	0.997
PF^b^	** − 29.29 (− 41.79; − 16.79)**	** < 0.001**	− 2.88 (.17.70; 11.94)	0.700
RP^b^	** − 25.57 (− 40.82; − 10.32)**	**0.001**	0.53 (− 19.38; 20.44)	0.958
BP^b^	** − 14.82 (− 25.62; − 4.03)**	**0.007**	2.63 (− 10.59; 15.84)	0.694
GH^b^	− 7.59 (− 16.86; 1.69)	0.108	− 0.26 (− 12.68; 12.17)	0.967
**SF36 mental component**^b^	− 6.16 (− 14.50; 2.18)	0.147	− 0.41 (− 11.83; 11.00)	0.944
VT^b^	** − 10.73 (− 19.05; − 2.41)**	**0.012**	− 3.81 (− 14.98; 7.36)	0.500
SF^b^	** − 15.49 (− 26.15; − 4.84)**	**0.005**	− 8.00 (− 22.39; 6.40)	0.273
RE^b^	** − 14.63 (− 28.63; − 0.64)**	**0.041**	− 3.61 (− 16.91; 24.14)	0.727
MH^b^	− 5.95 (− 14.27; 2.46)	0.165	− 1.60 (− 12.76; 9.56)	0.776
**Joint surgeries**^a^	**26.6 (3.62; 195.34)**	**0.001**	7.33 (0.82; 65.62)	0.075

*Legend*: *JIA* juvenile idiopathic arthritis, *bDMARD* biological disease-modifying antirheumatic drugs, *HAQ* Health Assessment Questionnaire, *SF36* Medical Outcomes Study 36-item Short Form, *SF36 PC* physical component of the Short Form 36, *SF36 MC* mental component of the Short Form 36, *PF* physical function, *RP* role limitations due to physical problems, *BP* intensity and discomfort caused by pain, *GH* general health, *VT* vitality, *SF* social function, *RE* role limitations due to emotional problems, *MH* mental health

^a^Logistic regression (regression coefficients presented as odds ratio (OR))

^b^Linear regression (regression coefficients presented as beta)

^c^Having JIA Persistent oligoarthritis as reference

^d^Adjustment for disease activity does not apply

The group of patients who started bDMARD after 5 years of disease onset had also a higher HAQ (in < 2 years group — median of 0 [IQR 0–0.25], 2–5 years group — median of 0 [IQR 0–0.09], > 5 years group — median 0.5 [IQR 0–1.25]; *p* < 0.001) and scored worse on the physical component of SF-36 (in < 2 years group — median of 56.2 [IQR 44.2–58.6], 2–5 years group — median of 49.5 [IQR 44.0–57.8] and > 5 years group — median of 40.2 [IQR 32.9–64.4]; *p* = 0.001). Also, in the mental domains of vitality and social function, the scores were likely to be worse if the bDMARD was started later than 5 years (median of 50 [IQR 40–69.4]; *p* = 0.008 and 75 [IQR 50–100]; *p* = 0.017, respectively) when compared to an earlier start (Table 2). These associations were independent of the JIA category or gender but not of disease duration or disease activity (Table 3).

Patients who started bDMARD after 5 years of disease onset also underwent joint surgery more often than those who started bDMARDs earlier (24.1% vs 2.2%, *p* < 0.001). The odds of having a joint surgery were increased (odds ratio (OR) = 26.6, 95% CI: 3.62–195.34; *p* = 0.001) if the patients started bDMARDs after 5 years of disease onset in an independent way of JIA category and gender but not of the disease duration or disease activity (Table 3).

## Discussion

The early start of biologic therapy in pediatric patients with JIA is crucial when the control of the disease with conventional DMARDs is not achievable [12, 21]. This is reflected in the more recent recommendations for JIA treatment where bDMARDs, according to a treat-to-target strategy, should be considered in an early phase of the disease, in order to achieve remission if this is not reached with cDMARDs [2, 22–24]. However, it is less clear what is the long-term impact of the timing of bDMARD initiation as an independent factor on JIA outcomes.

In our study, we found that only a minority of the patients started bDMARDs early in the disease course (14.4% in the first 2 years after disease onset). One possible reason for this observation is that more than half of these patients (53.3%) had the disease onset before the year 2000 and thus did not have access to bDMARDs during the first years of their disease course.

This adult JIA population had a predominance of polyarticular and ERA categories, which reflects the JIA population that prevails in adult rheumatology care [25]. The distribution of the JIA categories according to the 3 groups, showed for all of them a higher prevalence in the > 5 years group, even for the systemic-onset JIA where the bDMARDS are now recommended in the early stages of the disease and even in first-line [22]. However, also in this JIA category, more than half of the patients (55.9%) had the disease onset before the year 2000 which explains the distribution over the 3 study groups. Nevertheless, even in the subgroup of this cohort that had the disease onset after the year 2000, 41.6% started the bDMARD later than 5 years of disease onset. This is in line with other registries like Biker and Biobadaser, which showed that in 2000, only around 25% of patients received the first biologic at a pediatric age, with this percentage increasing linearly until reaching 65% in 2015 [8, 26].

To our knowledge, besides our study, only Minden et al. [8] analyzed the relation of an early versus late start of bDMARD in the JIA course and the likelihood of having a drug-free remission, full functional capability, and the need for joint surgery. Similarly to what was found in this previous study [8], in our cohort patients who started bDMARD after 5 years of disease onset were less likely to achieve remission off medication, when compared to the patients who started bDMARD earlier, irrespectively of the JIA category, gender, and disease duration. Up to 5 years we could not find any significant differences regarding the timing of an earlier start (less than 2 years and between 2 and 5 years). However, the evidence that a later bDMARD start decreases the chance of achieving remission off medication supports the concept of the “window of opportunity” that has been defended also for JIA and guides the treat-to-target strategy proposed for JIA [2].

As expected, given the fact the patients in our study who started bDMARD after 5 years of disease onset were older, had longer disease duration, and most of them had their disease onset before the biological era, these patients had more physical disability and worse scores regarding the physical component of SF-36, and vitality and social function evaluated by the mental component of the SF-36. In fact, the association between these worse results regarding physical disability and HRQoL and the timing of bDMARD initiation was shown to be independent of JIA category or gender but not independent of disease duration or disease activity, which could have more weight in these outcomes than the timing of bDMARDs initiation by itself. This is in line with several studies that had shown that disease duration and disease activity (especially pain) are important predictors for worse HRQoL and physical disability [27–29].

Recently data from BiKeR/JuMBO registers showed that patients who were refractory or intolerant to conventional treatment and started bDMARDs within the first two years of JIA onset had a significantly lower likelihood of requiring joint surgery [8]. Similarly, we found that the odds of having a joint surgery were increased if the patients started bDMARDs after 5 years of disease onset in an independent way of JIA category and gender. However, in our study, this association was not independent of the disease duration or disease activity that seem to be more important contributors to this outcome than the moment of bDMARD initiation.

Our study has some limitations. First, its cross-sectional design may not accurately estimate the evolution over time of disease activity and HRQoL in JIA patients. As this is a long-term study the effect of different treatment strategies might have also had an impact on these outcomes at different time points. Additionally, selection bias of the registry may overrepresent more severe cases and some categories of JIA, like the ones with polyarticular involvement, as many patients in remission could have been lost for follow-up and patients with milder disease could have been less motivated to be enrolled. Another factor is that the moment of starting the first cDMARD was not considered since most patients were enrolled in Reuma.pt at the start of a bDMARD therapy. The number of patients in each JIA category, grouped according to the timing of bDMARD initiation, was too small to allow the study of the impact of bDMARD start on the outcomes for each category.

This study’s strength lies in the large dimension of the adult JIA patient cohort, in its long follow-up, and in the fact that it reflects real-world evidence and clinical practice. This is one of the first studies to address the influence of the timing of biological treatment initiation on JIA long-term health-related outcomes.

## Conclusion

In conclusion, our results document that in JIA patients, the late start of bDMARDs increases the likelihood of having more physical disability and worse HRQoL and decreases the chances of achieving remission off medication in adulthood.

## Data Availability

Data is available upon request.

## Abbreviations

ACR: American College of Rheumatology
ACPA: Anti-citrullinated protein antibodies
ANA: Antinuclear antibodies
bDMARD: Biological disease-modifying antirheumatic drug
cDMARD: Conventional disease-modifying antirheumatic drugs
CID: Clinical inactive disease
CR: Clinical remission
HRQoL: Health-related quality of life
HAQ-DI: Health Assessment Questionnaire—Disability Index
HLAB27: Human leukocyte antigen B27
JADI-A: Juvenile Arthritis Damage Index – articular
JADI-E: Juvenile Arthritis Damage Index – extra-articular
JIA: Juvenile idiopathic arthritis
SF-36: Medical Outcomes Study 36-item Short Form
Reuma.pt: Rheumatic Diseases Portuguese Register
RA: Rheumatoid arthritis
RF: Rheumatoid factor
SpA: Spondyloarthritis
